# I'll Follow the Minority: The Effects of Sales Level on Purchase Intention of Self-expressive Products

**DOI:** 10.3389/fpsyg.2018.01135

**Published:** 2018-07-10

**Authors:** Xiaoyu Wan, Tingyi Wang, Jifei Wu

**Affiliations:** ^1^School of Economics and Management, Chongqing University of Posts and Telecommunications, Chongqing, China; ^2^Business School, Sun Yat-sen University, Guangzhou, China

**Keywords:** naive theory, self-expressive products, perceived self-image exclusivity, perceived face threat, purchase intention

## Abstract

The present study focuses on the naive theory of exclusivity (vs.popularity) triggered by the sales level of self-expressive (vs. functional) products and introduces perceived self-image exclusivity and perceived face threat to explain the effect of self-expressive products' sales levels on consumers' purchase intention. Specially, about 900 young people participated in four experiments, which used T-shirts, pillows, cups, fashion coats and heating blankets as experimental materials. Through four studies, it is found that consumers are more likely to choose self-expressive (vs. functional) products with low sales (vs. high sales) level. In addition, the paper presents a serial mediation effect of perceived self-image exclusivity → perceived face threat, which can explain the “I will follow the minority” effect of self-expressive products. Finally, the study presents the theoretical and practical significance and future research direction.

## Introduction

Imagine a consumer who is exploring the information of Amazon Best Sellers on amazon.com, intending to buy a coat and wet wipes there. How likely will he/she choose a coat with high sales level (namely, ranked as a best seller)? Will the choice differ from that concerning wet wipes?

Some online retail websites (e.g., American Amazon or China T-mall) show the sales level information (e.g., best seller rank or sales volume) of every product, which plays an important role during consumer decision making. Sales level, which reflects the number of buyers, will influence a consumer's choice of a product. This idea is generated by social naive theories because consumers often evaluate products or services based on common sense or naive theory (Raghunathan et al., 2006; Labroo and Mukhopadhyay, 2009; Yorkston et al., 2010; Deval et al., 2013). However, previous studies have found that consumers may hold contradictory naive beliefs about the same information (Deval et al., 2013; Steinhart et al., 2014). In some cases (e.g., buying functional products), consumers may choose products that many others like, driven by the naive theory of popularity, that is, preferring high sales level, and yet in other situations (e.g., buying self-expressive products), they will be attracted to products that few others are interested in, driven by the naive theory of exclusivity (Steinhart et al., 2014), that is, avoiding high sales level.

Follow previous research, sales level information's influence on the purchase intention of self-expressive products (vs. functional products) is driven by the naive theory of exclusivity (vs. popularity). This is because self-expressive products (vs. functional products) are better at expressing the image and self of their owners (Berger and Heath, 2007), while the high (low) sales indicates a more commonplace (exclusive) quality for both the self-expressive products and their owners (Machleit et al., 2000), which is a negative (positive) signal for consumers who seek self-image exclusivity through self-expressive products.

It is worth noting that previous research has shown that people consciously make different choices from others to highlight their uniqueness in the group (Chan et al., 2012), and the reason people choose scarce products is to maintain their uniqueness (Steinhart et al., 2014). However, this study argues that the exclusive preference of consumers for self-expressive products (vs. functional products) cannot be completely explained by the need for uniqueness; face also plays an important role in the consumer choice due to its close relationship with a person's desired self-image (Goffman, 1967; Litt et al., 2014). Thus, this study draws on the naive theory associated with self-expressive products to explore how the naive theory of exclusivity affects consumer choice considering sales level and reveals the underlying mechanism of face. Specifically, the present study addresses when consumers prefer to choose low (vs. high) sales level products and why they buy self-expressive products with low (vs. high) sales level. Findings revel that consumers prefer to choose or buy self-expressive (vs. functional) products with low sales and functional (vs. self-expressive) products with high sales; furthermore, the serial mediation effect of perceived self-image exclusivity → perceived face threat is proposed to explain the effect.

## Theoretical framework

### Naive theories in consumer behavior

Naive theories are defined as informal, common sense explanations that people use in their daily lives to influence the environment, and they often differ from formal, scientific explanations of what really happens (Furnham, 1988; Deval et al., 2013). Because the application and activation of naive theories requires minimal cognitive effort, and consumers often rely on naive theories for making inferences about marketing messages, products and services (Kardes et al., 2004), marketers often emphasize some product features that induce consumers' naive beliefs to optimize product marketing strategy (Lynn, 1992).

Previous research has shown that naive theories may conflict with each other and that consumers' assessments of products vary with the inferred rules triggered by prior priming (e.g., the popularity and exclusivity in a social context; Deval et al., 2013; Steinhart et al., 2014). Specifically, when following the naive theory of popularity, consumers will infer the interest of many others as a positive attribute (Steinhart et al., 2014). This phenomenon is similar to the “bandwagon” and the “As Seen on TV” effects (Hellofs and Jacobson, 1999; Powell and Prasad, 2010), which occur when consumers make a positive assessment of a product simply based on the number of people who have purchased or used it. In contrast, the naive theory of exclusivity suggests that the interest of many others may mean diminished product uniqueness (Lynn, 1992), leading consumers to think that the product is commonplace (Machleit et al., 2000); this is consistent with the “loss of exclusivity” mentioned in previous studies (Hellofs and Jacobson, 1999).

Following the reasoning of previous studies (Deval et al., 2013; Steinhart et al., 2014), activating a compelling naive theory can lead consumers to make purchase decisions by processing contextual cues. Moreover, as Deval et al. (2013) have showed, activation could be achieved by manipulating product popularity or exclusivity cues through presenting the number of people who are interested in a particular product. Further, as previous studies (Steinhart et al., 2014) have showed, the product itself (self-expressive vs. functional products) activates different naive beliefs, and the interaction of the contextual cues (interest of others) with the product further increases the capability of naive beliefs. However, the paper focuses on the naive theory of exclusivity activated by contextual cues (i.e., sales level information) and self-expressive products and examine whether and how the sales level of self-expressive products influences consumers' behavior.

### The impact of sales level on purchase intention

Sales level is an important contextual cue that reflects the consumer interest and market share of a product, and a higher sales level indicates that a product is more desirable in the market (He and Oppewal, 2017). Previous studies divided the sales level into higher and lower levels judging by how many people had bought or owned a product (He and Oppewal, 2017). Moreover, most previous studies demonstrated high sales level as a popularity cue, holding that a high sales level means that more people liked the product and had bought or owned the product; that is, the product was more popular in society than another product with a lower sales level (Wu and Lee, 2016). However, low sales level (namely, low market share) is a signal that the product has an exclusive image (Hellofs and Jacobson, 1999), meaning that only a few people own the same product, and so it is closely related to product uniqueness (Tian et al., 2001). For what role the sales level plays in consumer purchase, studies argue that it would positively influence people's choices through perceived product popularity (He and Oppewal, 2017) and that it would have different influences when the product was purchased for oneself or others (Wu and Lee, 2016); in particular, it would be a negative signal concerning a self-expressive product and a positive signal concerning a functional product (Steinhart et al., 2014).

This study focuses on the impact of sales level on purchase intention of self-expressive vs. functional products. Functional products and self-expressive products were distinguished according to the extent to which a product category is believed to signal the status of the owner, that is, the status signaling capability (SSC; Wang and Wallendorf, 2006; Chan et al., 2012. Usually, a self-expressive (functional) product is one can (cannot) signal the status or identity of the owner significantly (Chan et al., 2012; Steinhart et al., 2014). Previous studies have showed that a product was a part of the consumer, and people used products to express their identities and tastes (Berger and Heath, 2007). However, different products showed different capabilities to signal owners' status or identities, and so individuals used to use certain types of products to achieve their self-expression (Belk, 1981). Indeed, some products, that is, symbolic products (e.g., a T-shirt), rather than products that are more functional and less self-expressive (e.g., a stereo system; Shavitt, 1990), more easily communicated information about their owners (Escalas and Bettman, 2005). Overall, previous studies considered self-expressive products and functional products as two opposing product types (Steinhart et al., 2014), and these studies held that it was the status signaling capability rather than the performance of self-expressive products was important, on the contrary, it was the performance rather than the status signaling capability of functional products was important (Berger and Heath, 2007; Steinhart et al., 2014). Specifically, self-expressive products, which tend to include scarce and differentiated products (Tian et al., 2001; Steinhart et al., 2014); e.g., a unique, customized product or a product that could not be owned by others at the same time), possess self-expressive features, and an individual's consumption of them depends more on the personal or social meaning of the products than their functional utility (Berger and Heath, 2007). In contrast, a functional product is an essential, utilitarian tool that enables the owner to achieve a goal or complete a practical task (Wertenbroch and Dhar, 2000).

Besides, it is worth noting that this study just discusses the difference between unbranded self-expressive products and unbranded functional products, and the influence of famous brand on status signaling capability of products is beyond the scope of this study. The research avoided arousing product branding in experiments because a famous brand will increase the sales of products because people think that the consumption of the products with famous brand is a symbol of status.

According to previous studies, consumers' evaluation of functional products was often triggered by the naive theory of popularity. Consumers followed most people's judgement because they held that the wisdom of the majority cannot be wrong (Steinhart et al., 2014); therefore, they had a significantly more positive evaluation of the functional products with high vs. low level of sales. However, it is important to note that self-expressive products evoke the naive theory of exclusivity. Consumers' evaluations of self-expressive products, in contrast to evaluations of functional products, are predicted to be enhanced when few rather than many people own such products (Steinhart et al., 2014); that is, consumers' assessment of self-expressive products will be more positive when the sales level is low rather than high because a low sales level implies a scarcity and exclusivity of the products. Therefore, the following hypothesis is proposed:

*H1: Low vs. high level of sales will result in more purchase intention of self-expressive vs. functional products*.

### The serial mediation effect of perceived self-image exclusivity and perceived face threat

Self-image was treated as the actual self-concept, i.e., as a perception of oneself (Bellenger et al., 1976; Sirgy, 1982). One's self-image was influenced by his or her personality and image. Impression management theory proposed that people try to control how others perceive them (Leary and Kowalski, 1990); this study regarded exclusivity (Steinhart et al., 2014) as a factor that should be considered. Perceived self-image exclusivity, in the present study, was defined as the degree to which one infers that others share the same image, and the lower the perceived self-image exclusivity is, the more likely one's image will be similar to others'.

Previous research showed that, as consumers' concerns for exclusivity increased, the products' increased market share reduced the product evaluations (Hellofs and Jacobson, 1999). Hence, from a theoretical perspective, the interest of few others elevates product evaluation, as consumers rationalize that the product is not accessible to everyone (Steinhart et al., 2014); that is, the owners' self-image of the product is exclusive to some degree. In the present study, sales level is proposed as another important factor leading to exclusivity; a low (vs. high) sales level represents the product's exclusivity (vs. popularity) and scarce (vs. commonplace) image (Hellofs and Jacobson, 1999; He and Oppewal, 2017), but the perception of self-image exclusivity varies between the consumption of self-expressive and functional products. Because the symbolic characteristics of self-expressive (vs. functional) products match consumers' inner needs of image management and social identity (Whan Park, 1986), the consumption of self-expressive (vs. functional) products can bring social or individual meanings to individuals. Therefore, when considering a self-expressive product with low (vs. high) sales level, that is, an exclusive (vs. popularity) product, individuals will happily consider (vs. hardly consider) their self-image with the product as exclusive. In contrast, the consumption of functional products mainly meets the needs of quality and utility. Previous studies showed that consumers were mainly concerned about the popularity of functional products (Steinhart et al., 2014; Wu and Lee, 2016) to infer the quality and value; therefore, the product's exclusivity would not be associated with self-image. In conclusion, individuals usually hold a belief about self-expressive (vs. functional) products that high sales level indicates a commonplace self-image.

Face was defined as the “positive social value a person effectively claims for himself by his or her self-presentation” (Goffman, 1967). However, face threat is a situation that occurs when a person's desired image is challenged or undermined (Goffman, 1967; Cupach and Metts, 1994), and it can be generated by the self (e.g., one found himself/herself wearing the same coat as someone else) or others (e.g., one's coat got laughed at; Cupach and Metts, 1994). Following the definition of face threat from previous studies (Goffman, 1967; Cupach and Metts, 1994), the present study defined perceived face threat as an inner perception or inference about how much a situation will challenge or undermine a person's desired image.

Face represents a supportive social self-image (Litt et al., 2014); therefore, when others' consumption behavior or potential evaluation in the environment impedes the individual from maintaining his or her own social self-image, face threat is generated. As predicted by this study, a self-expressive product with high (vs. low) sales level triggers less (vs. more) perceived self-image exclusivity; that is, the user of a product considered commonplace might sense negative evaluations about his or her image, which is contrary to individuals' desired self-image, that is, in contrast to his or her targeted self-presentation. Thus, face threat is generated. In contrast, the consumption of functional products hardly concerns whether the product can achieve social self-image, and so the need for face is not significant, and the popularity from a high sales level will not be associated with less face for consumers, that is, face threat. Individuals would try to avoid face threat or save face through their own behavior because when experiencing face threat with different levels of severity (Petronio, 2002; Litt et al., 2014; Wohn and Spottswood, 2016), caused by imagining or perceiving negative evaluations from others, individuals would produce a series of negative emotions or reactions (Wohn and Spottswood, 2016). Thus, the present study argued that the lower perceived self-image exclusivity associated with high sales level will significantly increase perceived face threat and ultimately reduce the purchase intention of the self-expressive products, rather than of functional products with high sales level.

**Figure 1:**
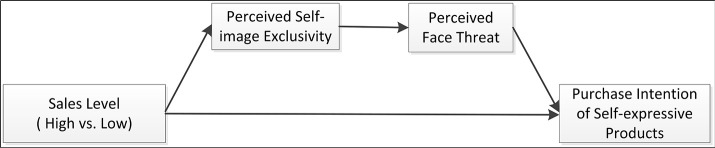
Theoretical model.

In conclusion, individuals usually hold the belief concerning self-expressive (vs. functional) products that high sales means commonplace self-image and negative evaluations from others. Therefore, the following hypothesis is proposed:

*H2: The effect of sales level on individuals' purchase intention of a self-expressive (vs. functional) product is serially mediated by perceived self-image exclusivity and perceived face threat (visualized as Figure 1)*.

### Overview of studies

Theoretical propositions were tested in a series of studies. First, Study 1A, conducted under the condition of accepting prizes, explored whether low (high) level of sales will result in more self-expressive (functional) products' choices. Second, Study 1B nearly replicated the experiment in Study 1A, but to enhance the generalizability of the present study, cups described as either a self-expressive or a functional product were the experimental stimuli. Afterwards, in the condition of online shopping, Study 2 tested the proposed serial psychological mechanisms via perceived self-image exclusivity and perceived face threat of self-expressive (vs. functional) products. Finally, with discounting the belief that “high sales means commonplace and negative evaluations from others,” which is the cause of the serial mediation, Study 3 tested whether the effect of different sales level on self-expressive products would disappear. Several unbranded products were used as materials to exclude the impact of product brand on the consumer purchase intention.

All participants gave their informed consent before Study 1A, Study 1B, Study 2, and Study 3. All of studies were conducted under the approval of the Academic Committee of the Department of Economics and Management at Chongqing University of Posts & Telecommunications.

### Study 1A

Study 1A was conducted to examine the basic hypothesis that low vs. high level of sales will result in more choice of self-expressive vs. functional products.

### Methods

#### Participants and design

Participants (*N* = 160, *M*_age_ = 21.33, *SD*_age_ = 0.84, 55% female), who were asked to complete an online questionnaire about their perceptions and preference about activity prizes, were undergraduate business students at a southwest university of China who participated for course credit. Participants were randomly assigned to one of the two experimental conditions (sales level: high or low).

#### Stimuli and procedure

T-shirts and pillows were selected as self-expressive and functional products in the Study 1A. A pre-test was used to confirm a pair of self-expressive and functional products. Fifty-two participants (*M*_age_ = 21.46, *SD*_age_ = 0.83, 56% female) were exposed to a pair of products on mobile screen: T-shirts and pillows. They were then asked to rate their agreement with the following four statements of the scale about the status signaling capability (Wang and Wallendorf, 2006): “T-shirts (or pillows) can convey one's personality to the people around him/her,” etc., (1 = *strongly disagree*, 7 = *strongly agree*), Cronbach's α = 0.85 (see Appendix A). Usually, the higher (lower) the average score was, a product tended to be a self-expressive (functional) product. Specially, a product which scored 1 (7) point of each questions was completely a functional (self-expressive) product. In the pre-test, participants perceived that T-shirts had significantly stronger capability to signal status than pillows (*M*_T−shirts_ = 5.4, *M*_pillow_ = 3.6, *p* < 0.001). Therefore, Study 1A selected T-shirts and pillows as self-expressive and functional products.

All instructions and questionnaires were presented via desktop. Each participant was exposed to a scenario assuming that they had won third prize in an activity, and the optional prizes were pillows and T-shirts all priced at $22. To manipulate sales level, participants in the low level of sales condition were told that two kinds of prizes had been chosen by very few people. In the high level of sales condition, participants were told that two kinds of prizes had been chosen by a large number of people. Then, all participants were asked to make a choice between the two prizes. Finally, participants rated their agreement with the statements which was the perception of sales level “Many others are likely to own this product” and “Few others are likely to own this product” (Steinhart et al., 2014) on a 7-point scale from 1 (*strongly disagree*) to 7 (*strongly agree*).

### Results

Fourteen of one hundred sixty participants were deleted for either inconsistent answers or incomplete answers. One hundred and forty six valid data points were used (*N*_high_ = 73, *N*_low_ = 73).

#### Manipulation check

Participants in the high sales level condition more strongly agreed with the statement that many others were likely to own each product (*M*_high_ = 4.92) than participants in the low sales level condition [*M*_low_ = 3.41; *t*_(144)_ = 3.73, *p* < 0.001]. In addition, participants in the high sales level condition agreed significantly less with the statement that few others were likely to own each product [*M*_high_ = 2.87, *M*_low_ = 4.25; *t*_(144)_ = 3.65, *p* < 0.001]. The manipulation of sales level was successful.

#### Choice

**Figure 2:**
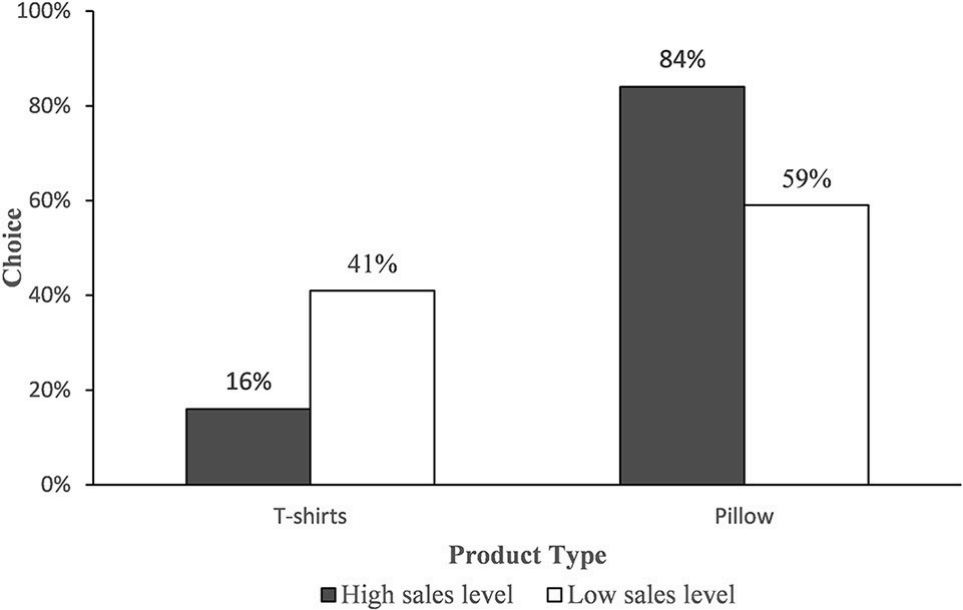
Choice of T-shirts.

The results of the Chi-square test showed that participants in the low level of sales condition were more likely to choose a self-expressive product than participants in the high level of sales (*M*_low_ = 41%, *M*_high_ = 16%, χ^2^ = 10.830, *p* < 0.005). Conversely, participants were more likely to select functional products with a high sales level (*M*_high_ = 84%, *M*_low_ = 59%, χ^2^ = 10.830, *p* < 0.005) (see Figure 2), in support of H1. Furthermore, there was no significant difference in the influence of gender on choice (χ^2^ = 0.343, *p* = 0.558 > 0.05).

### Discussion

The results of Study 1A showed that participants were more willing to choose self-expressive (vs. functional) products at low (vs. high) sales levels. However, it might be considered that the two products in Study 1A were so different in terms of use that participants' valuation and a real need for them would result in bias. Therefore, in Study 1B, participants were exposed to two similar products.

### Study 1B

Study 1B also examined whether consumers choose different products (described as functional or self-expressive) considering different sales levels. Individuals' choice of self-expressive products were expected to more likely correspond with the low sales level.

### Methods

#### Participants and design

Participants (*N* = 163, *M*_age_ = 21.5, *SD*_age_ = 0.85, 53% female), who were invited to complete an online questionnaire about their preference and perceptions about a birthday gift, received compensation of 10 RMB. All participants were randomly assigned to one of two conditions (sales level: high or low).

#### Stimuli and procedure

Cups were selected as stimulus in this study: adiabatic cup (functional product) or distinctive cup (self-expressive product). In a pre-test with 35 participants (*M*_age_ = 20.33, *SD*_age_ = 0.74, 46% female), participants were invited to use a 7-point scale same as Study 1A to rate their agreement with the products' capability to signal status (Wang and Wallendorf, 2006; Chan et al., 2012), *Cronbach's* α = 0.83. In the pre-test, participants perceived that the distinctive cup had a significantly stronger capability to signal status than the adiabatic cup (*M*_distinctivecup_ = 5.2, *M*_adiabaticcup_ = 4.1, *p* < 0.001); the selection of two products was suitable.

All instructions and questionnaires were presented via desktop as Study 1A. Each participant was exposed to a scenario: “your birthday is coming, and one of your good friends prepared to give you a birthday gift (a cup) and told you to use it by yourself. He/she allows you to choose one of two (either a distinctive cup or an adiabatic cup, with same price and same specifications).” Participants exposed to high (low) sales level were told that both cups were used by many (few) people. Then, participants chose one from two cups. Finally, participants' perception of sales level were measured on a 7-point scale as Study 1A.

### Results

Eight of one hundred sixty three participants were deleted for either inconsistent answers or incomplete answers. One hundred and fifty five valid data points were used (*N*_high_ = 79, *N*_low_ = 76).

#### Manipulation check

Participants exposed to the high sales level more strongly agreed with the statement that many others were likely to own each product (*M* = 5.3) than those exposed to the low sales level condition [*M* = 2.13; *t*_(153)_ = 3.13, *p* < 0.001]; In addition, participants exposed to the high sales level condition agreed significantly less with the statement that few others were likely to own each product [*M*_high_ = 2.55, *M*_low_ = 5.15; *t*_(153)_ = 3.12, *p* < 0.01]; the manipulation of sales level was successful in Study 1B.

#### Choice

**Figure 3:**
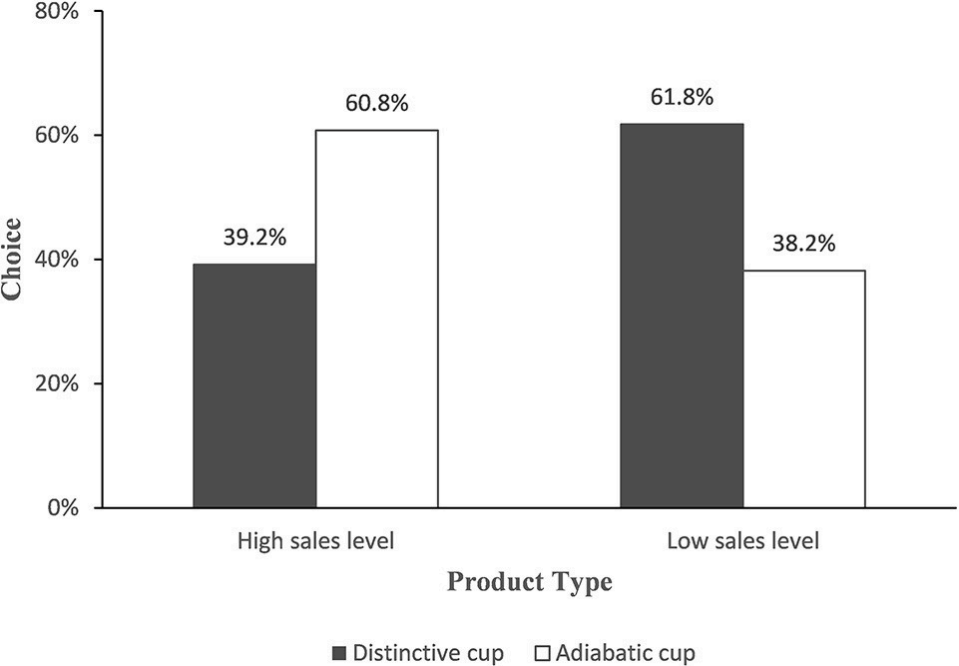
Choice of distinctive cup.

A Chi-square test of participants' reported choice showed that consumers were more likely to choose the distinctive cup with a lower sales level (61.8%, χ^2^ = 7.915, *p* < 0.01) compared to the one with a high sales level (39.2%). Conversely, individuals were more likely to select an adiabatic cup with a high sales level (*M*_high_ = 60.8%, *M*_low_ = 38.2%, χ^2^ = 7.915, *p* < 0.01, (see Figure 3). That is, in line with Study 1 and H1, individuals had a stronger intention to accept self-expressive products (vs. functional products) with low (vs. high) sales level. Furthermore, there was no significant difference in the influence of gender on choice (χ^2^ = 0.008, *p* = 0.927 > 0.05).

### Discussion

Study 1B replicated and strengthened the results of Study 1A. Different from Study 1A, Study 1B used products that were more similar than those in Study 1A as stimulus and presented a scenario of accepting a birthday gift. As expected, participants' choice did not differ from that in Study 1A, in supporting of H1 again. However, it might be doubted that participants have to choose between a self-expressive product and a functional product in either high or low sales level condition, which is different from the online purchase process, where they can choose one product with various sales levels.

### Study 2

In Study 2, the underlying mechanisms behind the effect of sales level on self-expressive products' purchase intention were explored initially. Specifically, how exposure to high (vs. low) sales level of self-expressive (vs. functional) products influences a consumer's purchase intention were examined. It was expected that consumers' purchase intentions to be higher when exposed a low (vs. high) sales level of self-expressive (vs. functional) products (H1). Moreover, perceived self-image exclusivity and perceived face threat were expected to serially mediate the effect of sales level on consumers' purchase intentions of self-expressive products (H2).

### Methods

#### Participants and design

Participants (*N* = 224, *M*_age_ = 19.4, *SD*_age_ = 1.21, 51% female), who received compensation of 10 RMB, were invited to complete an online questionnaire about their purchase intention and perceptions about two products they would need in the coming winter. Study 2 used a two factors (sales level: high vs. low) ^*^ (product type: self-expressive product vs. functional product) between-subjects design. All participants were randomly assigned to one of four conditions.

#### Stimuli and procedure

Fashion coats and heating blankets were selected as self-expressive products and functional products, respectively. In a pre-test with 53 participants (*M*_age_ = 21.02, *SD*_age_ = 1.13, 48% female), a same measurement of the products' capability to signal status as Study 1A & 1B was repeated to successfully demonstrate that the fashion coat (heating blanket) was an expressive (a functional) product (*M*_fashion coat_ = 5.4, *M*_heating blanket_ = 2.9, *p* < 0.001). Furthermore, another stimulus was a picture of snow, which can enhance the desire to buy the above products.

All instructions and questionnaires of four conditions were presented via desktop as Study 1A and Study 1B. Each participant in the conditions of self-expressive products or functional products was exposed to the picture of snow and a scenario stating: “*winter is coming; you decide to buy a fashion coat (a heating blanket) for yourself, so you start to choose*.” Then, participants exposed to high (low) sales level conditions expected to find a fashion coat or a heating blanket with good style, price and material, etc., in the list of products with high (low) sales level.

#### Measures

After exposure to the scenario, participants in each condition responded to measures about their purchase intentions and perception of different products with different sales levels. The following dependent measures were used: (1) purchase intention (Dodds et al., 1991): How likely would you be to buy the fashion coat (heating blanket)? (1 = *not at all*, 7 = *very much*); (2) perceived self-image exclusivity (made some changes from Steinhart et al., 2014): you infer that others around you will not have the same image as you when using the fashion coat (heating blanket). (1 = *strongly disagree*, 7 = *strongly agree*); (3) perceived face threat: seven items of the severity of face threat scale from Litt et al. (2014) were adopted and changed slightly: “using the fashion coat (heating blanket) will make me feel awkward,” etc., (1 = *strongly disagree*, 7 = *strongly agree*), Cronbach's α = 0.904 (see Appendix A); (4) perceived uniqueness was measured to exclude the influence of uniqueness on the underlying mechanism: the extent to which others think the product reflects its user's uniqueness (1 = *very low*, 7 = *very high*); (5) sales level perception was measured as in Study 1A and Study 1B.

### Results

Sixteen of two hundred and twenty four participants were deleted for either inconsistent answers or incomplete answers. Two hundred and eight valid data points were used (*N*_high and self-*expressive*_ = 50, *N*_high and functional_ = 54, *N*_low and self-*expressive*_ = 51, *N*_low and functional_ = 53).

#### Manipulation check

Participants exposed to the high sales level more strongly agreed with the statement that many others were likely to own each product (*M* = 5.6) than those exposed to the low sales level condition [*M* = 1.12; *t*_(206)_ = 2.66, *p* < 0.01]; participants exposed to the high sales level condition agreed significantly less with the statement that few others were likely to own each product [*M*_high_ = 1.56, *M*_low_ = 5.23, *t*_(206)_ = 2.95, *p* < 0.01]; the manipulation of sales level was successful in Study 2.

#### Purchase intentions

**Figure 4:**
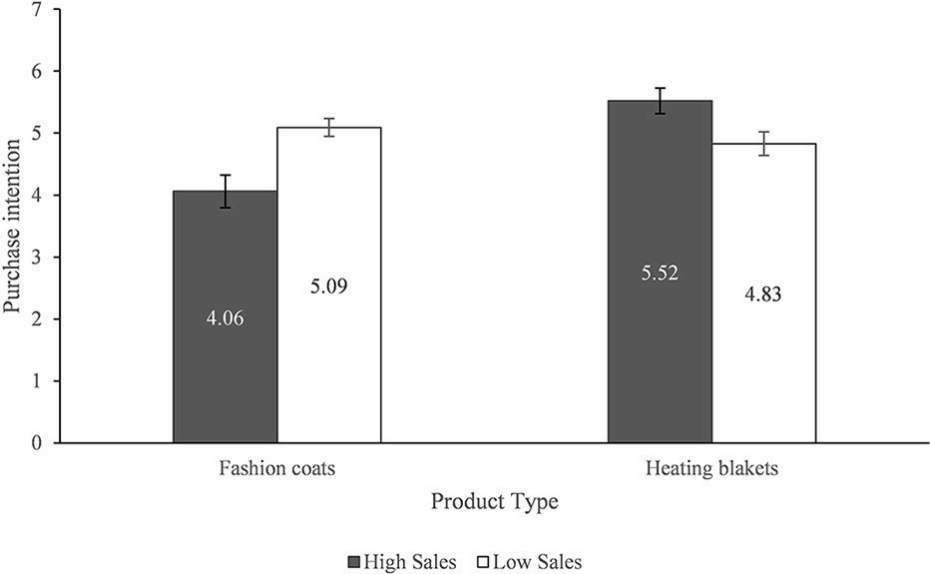
Purchase intention of fashion coats.

We initially conducted a 2 × 2 between-subject ANOVA analysis, with two sales level (high or low) and two product types (self-expressive or functional). The main effect of sales level on purchase intention was not found to be significant, *F*_(1, 204)_ = 0.180, *p* = 0.671 > 0.05, but the interaction effect between sales level and product types was significant, *F*_(1, 204)_ = 15.638, *p* < 0.001. Specifically, an ANOVA analysis showed that the effect of sales level on purchase intentions of self-expressive products was significant, *F*_(1, 99)_ = 11.896, *p* < 0.005. Participants expressed higher intention (*M* = 5.1) to purchase the fashion coat with low sales level but lower intention (*M* = 4.1) to buy that with high sales level. In contrast, additional ANOVA analysis revealed that, for functional products, participants were significantly more likely to buy a heating blanket with a high sales level (*M* = 5.52) than one with a low sales level (*M* = 4.03), *F*_(1, 105)_ = 7.104, *p* < 0.01, (see Figure 4). Thus, H1 was supported again. Furthermore, there was no significant difference in the influence of gender on purchase intention of both fashion coat, *F*_(1, 102)_ = 0.466, *p* = 0.496 > 0.05, and heating blanket, *F*_(1, 102)_ = 0.002, *p* = 0.965 > 0.05.

#### Serial mediation test

**Figure 5:**
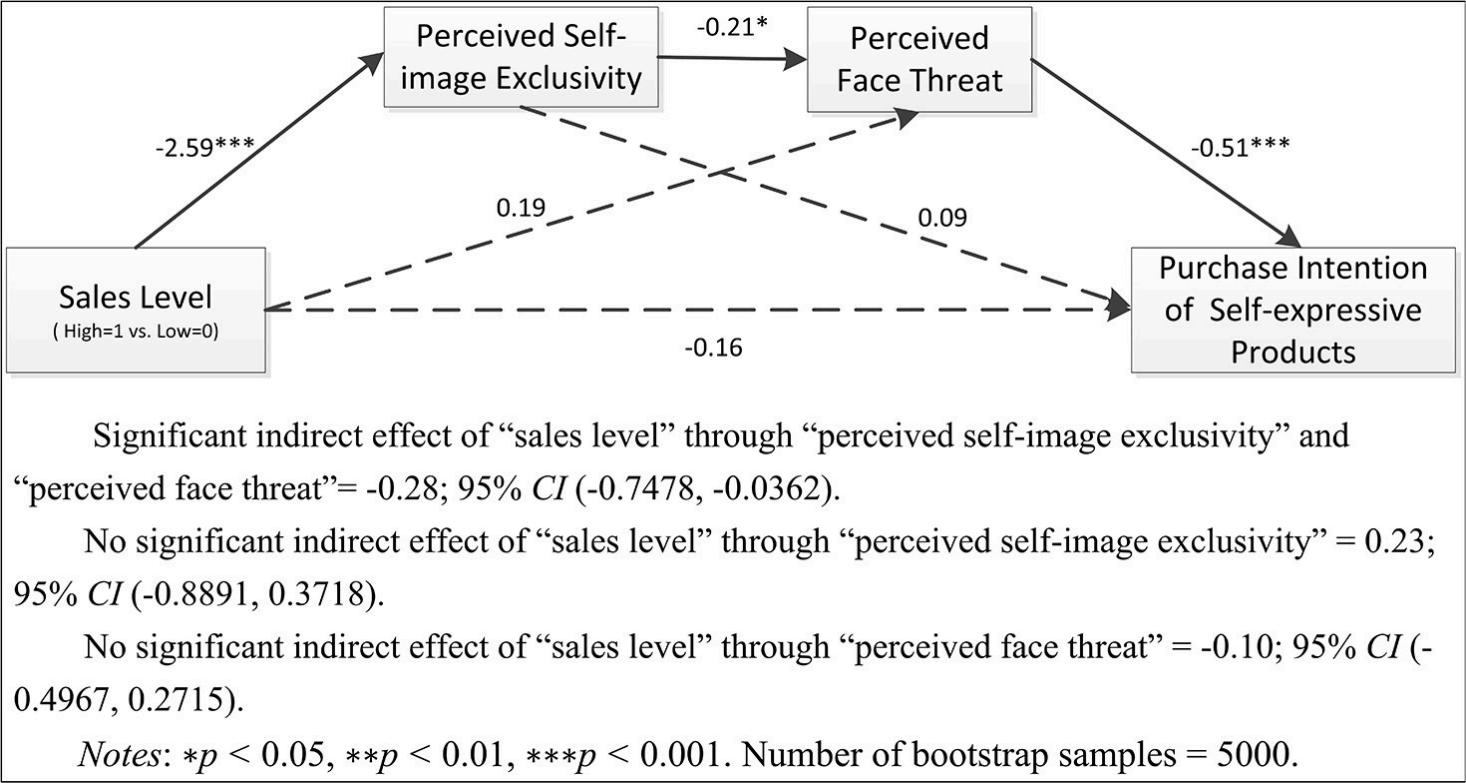
Indirect effect of sales level on purchase intention of self-expressive products.

To provide evidence for the underlying psychological mechanisms, analysis followed the steps suggested by Hayes' PROCESS procedure (Hayes, 2013). Respectively, mediation test on the conditions of self-expressive products' and functional products' purchase intention were conducted. Perceived uniqueness, gender and age were used as covariates throughout all the analysis. As shown in Figure 5, findings reveal that for self-expressive products' purchase, the serial mediation effect of perceived exclusivity → perceived face threat explains the negative impact of high level sales on products' purchase intentions (*B* = −0.28, bootstrapped 95% *CI*: −0.7478, −0.0362). Given that the direct effect of sales level on purchase intention is not significant (*B* = −0.16, bootstrapped 95% *CI*: −1.0557, 0.7292), it can be concluded that the serial effect of perceived exclusivity → perceived face threat fully mediates the effect of sales level on purchase intention for self-expressive products' purchase (H2 supported). In other words, for self-expressive products' purchase, low (vs. high) level sales significantly increases perceived self-image exclusivity (*M*_low_ = 5.13, *M*_high_ = 2.24; *t* = −12.6, *p* < 0.001), which leads to weaker (vs. stronger) perceived face threat (*M*_low_ = 3.51, *M*_high_ = 4.83; *t* = 4.8, *p* < 0.001), and finally increases (vs. decreased) purchase intentions.

However, for functional products' purchase, the serial mediation effect of perceived exclusivity → perceived face threat was not significant (*B* = −0.0014, bootstrapped 95% CI: −0.1219, 0.0914), as expected. That is, for functional products, low (vs. high) sales level neither significantly increases perceived self-image exclusivity (*M*_low_ = 5.31, *M*_high_ = 4.32; *t* = −6.335, *p* > 0.05), nor yields significantly different perceived face threat (*M*_low_ = 2.44, *M*_high_ = 2.83; *t* = −1.58, *p* > 0.05) considering two sales levels. Therefore, sales level could not influence consumers' purchase intention by the serial mediation effect of perceived exclusivity → perceived face threat.

### Discussion

Study 2 shows that individuals respond to low sales level with a more positive purchase intention when purchasing self-expressive products. Conversely, when purchasing functional products, individuals exhibit more positive purchase intention when sales level is high (vs. low) (supporting H1 once again). In addition, ruling out perceived uniqueness as the alternative explanation, it was demonstrated that, when purchasing self-expressive products with low (high) sales level, increasing (decreasing) perceived self-image exclusivity drives weaker (stronger) perceived face threat, which in turn influences consumers' purchase intentions. However, when purchasing functional productions, the mechanism of self-expressive products' sales level on purchase intention is not a concern. H2 was supported.

However, it might be doubted that when purchasing self-expressive products, there may be other explanations in addition to perceived self-image exclusivity, perceived face threat. In Study 3, H2 were tested by discounting the belief that “high sales means commonplace and negative evaluations from others,” which the paper discussed as the cause of the serial mediation.

### Study 3

Study 3 aimed once again to verify the mechanism underlying the effect of sales level of self-expressive products on purchase intention, using moderation approaches. If consumers activate their perceived self-image exclusivity and perceived face threat to judge a self-expressive product with low (high) sales level, then discounting the belief of “high sales means commonplace and negative evaluations from others” will attenuate the effect of low (high) sales level on increasing (decreasing) consumers' purchase intention of self-expressive products. To examine this prediction, participants' belief were manipulated through a priming task. Moreover, participants' activation of the belief were measured after the priming task and examined whether this belief mediated the effect on product purchase intention.

### Methods

#### Participants and design

Participants (*N* = 186, *M*_age_ = 19.8, *SD*_age_ = 1.22, 49% female), who received compensation of 10 RMB, were invited to complete an online questionnaire about their purchase intention and perceptions about a fashion coat they would need in the coming winter. All participants were randomly assigned to one of three conditions (sales level and discounting belief: high level and baseline vs. low level and baseline vs. high level and discounting belief).

#### Stimuli and procedure

On the one hand, consistent with Study 2, fashion coat was the self-expressive product and a snowy picture was the stimuli in this study. On the other hand, a fictitious research report titled “*High Sales Level Is Not a Big Deal*” was another stimuli. The report used approximately 300 words to describe a research study showing that even if someone bought a self-expressive product with high sales level, it is hard to find someone using the same product because of the large population, and notably, two people who use the same self-expressive product would not be negatively evaluated by others. The key message of the report was that empirical evidence does not support a significant association between high sales level and low self-image exclusivity and stronger face threat. A pre-test (*N* = 51, 45% female) confirmed that participants who read the “*High Sales Level Is Not a Big Deal*” report, compared with those who just read a weather report in the baseline condition, were less likely to think high sales level of self-expressive products was strongly associated with low self-image exclusivity and high face threat.

All instructions and questionnaires were presented via desktop as Study 2. First, participants were invited to complete a reading task, which was reading a report either about weather (high level and baseline, low level and baseline conditions) or “*High Sales Level Is Not a Big Deal*” (high level and discounting belief condition). Second, participants were guided to summarize the main idea of the report. Then, participants with the condition of high sales levels and participants with the condition of low sales level were presented with the same snowy picture and scenario of searching for fashion coats as used in Study 2, respectively.

#### Measures

After exposure to the scenario, participants in each condition responded to measures about their purchase intentions and perception toward fashion coats with different sales levels, just as the participants in Study 2 did. The following dependent variables were measured: (1) purchase intention; (2) perceived self-image exclusivity; (3) perceived face threat; (4) perceived uniqueness; (5) sales level perception.

### Results

Ten of one hundred eighty six participants were deleted for either inconsistent answers or incomplete answers. One hundred seventy six valid data points were used (*N*_high and baseline_ = 53, *N*_low and baseline_ = 59, *N*_high and discount_ = 64).

#### Manipulation check

Participants exposed to the high sales level with both baseline and discount belief conditions more strongly agreed with the statement that many others were likely to own the fashion coat (*M* = 5.3) than those exposed to the low sales level condition (*M* = 1.27; *t*_(174)_ = 2.11, *p* < 0.01); in addition, they agreed significantly less with the statement that few others were likely to own the fashion coat [*M*_high_ = 1.89, *M*_low_ = 5.41; *t*_(174)_ = 1.96, *p* < 0.001]; the manipulation of sales level was successful in Study 3.

#### Purchase intention

We initially conducted an ANOVA analysis among three conditions (high and baseline, low and baseline, high and discount). The differences on purchase intention among three conditions were found to be significant, *F*_(2, 173)_ = 13.005, *p* = <0.001. Specially, an ANOVA analysis between two conditions (high and baseline, low and baseline) showed that the effect of sales level on the purchase intention of fashion coat was significant, *F*_(1, 110)_ = 8.890, *p* < 0.005. Participants exposed to a low sales level had a higher intention (*M* = 5.1) to buy the coat, but those exposed to a high sales level had a lower intention (*M* = 4.23) to buy it. In line with Study 2, this further supported H1. Furthermore, there was no significant difference in the influence of gender on purchase intention of fashion coat, *F*_(1, 110)_ = 1.007, *p* = 0.302 > 0.05.

#### Serial mediation test

To further provide evidence for the serial mediation of Study 2, analysis following the steps suggested by Hayes' PROCESS procedure (Hayes, 2013) to test the serial mediation effects of self-expressive products (here a fashion coat). Perceived uniqueness, gender and age were used as covariates throughout all the analysis as Study 2.

First, an analysis between high sales level (coded as 1) and low sales level (coded as 0), both with baseline belief, (see Model 1 of Appendix B), showed that the serial mediation effect of perceived self-image exclusivity → perceived face threat explains the negative impact of high (vs. low) sales level on products' purchase intentions (*B* = −0.27, bootstrapped 95% *CI*: −0.7130, −0.0526), H2 was further supported. In other words, low level (vs. high level) sales significantly increase perceived product exclusivity (*M*_low_ = 5.15, *M*_high_ = 2.34; *t* = −12.01, *p* < 0.001), which leads to weaker (vs. stronger) perceived face threat (*M*_low_ = 3.74, *M*_high_ = 4.77; *t* = 4.17, *p* < 0.001), and finally drives increased (vs. decreased) purchase intentions.

Second, an analysis comparing high sales level with the baseline condition (coded as 1) and high sales level with the discounted belief condition (coded as 0) showed that the serial mediation effect of perceived self-image exclusivity → perceived face threat explains the negative impact of high level sales with the baseline belief (vs. discount belief) on products' purchase intentions (*B* = −0.1211, bootstrapped 95% *CI*: −0.3292, −0.0304) (see Model 2 of Appendix B). Thus, it can be concluded that the manipulation of the discounted belief is legitimate. The high sales level with discounted belief (vs. baseline belief) significantly increases perceived self-image exclusivity (*M*_discount belief_ = 3.66, *M*_baseline belief_ = 2.34; *t* = −4.77, *p* < 0.001), which leads to weaker (vs. stronger) perceived face threat (*M*_discount belief_ = 3.80, *M*_baseline belief_ = 4.77; *t* = 3.89, *p* < 0.001), and finally drives increased (vs. decreased) purchase intentions.

Third, an analysis comparing the conditions of low sales level with baseline belief (coded as 0) and high sales level with discount belief (coded as 1) revealed that not only was the serial mediation effect of perceived self-image exclusivity → perceived face threat not significant (*B* = −0.0309, bootstrapped 95% CI: −0.1276, 0.0013) (see Model 3 of Appendix B), and the direct effect of sales level on purchase intention was also not significant (*B* = 0.2460, bootstrapped 95% *CI*: −0.2886, 0.7805). That is, the discounted belief with high sales level condition increased perceived self-image exclusivity, which weakened perceived face threat and further increased purchase intention. This is similar to the effect on products with a low sales level condition (with the baseline belief).

In conclusions, findings revealed that when discounting the belief which causes the perceived self-image exclusivity and face threat, the effect of different sales level on self-expressive products' purchase intention would not significant. Moreover, consumers in discounted belief conditions, rather than those in baseline conditions, would have significant stronger intentions to buy self-expressive products with high sales level. Together, the results of Study 3 provided more powerful and favorable evidence for H2.

### Discussion

Again, Study 3 demonstrated that for self-expressive products, consumers hold stronger intention to buy given a low sales level. Furthermore, by priming a discounted belief, Study 3 provided powerful evidence of the serial mediation consistent with Study 2 for self-expressive products, which supported the proposed mechanism for the effect of self-expressive products' sales level on consumer purchase intention in H2.

## General discussion

This study demonstrated a link between self-expressive products and naive theory of exclusivity in different contexts of buying products and explored the connection from the perspective of face threat. That is, exposing individuals to a self-expressive (vs. functional) product triggers the naive theory of exclusivity (vs. popularity), and a low (vs. high) sales level will further activate the naive theory of exclusivity (vs. popularity). This supports the premise concerning self-expressive products with different sales level that consumers are more likely to choose a self-expressive (vs. functional) product in low (vs. high) sales level condition (Studies 1A and 1B), and that when purchasing a self-expressive (vs. functional) product, consumers are more likely to buy one with low (vs. high) sales level (Studies 2 and 3). Notably, unlike previous studies, which hold consumers' self-perceptions of uniqueness as a main factor of self-expressive products' purchase and contextual cues (Steinhart et al., 2014), this study, ruling out the influence of perceived uniqueness, found that the serial mediation effect of perceived self-image exclusivity → perceived face threat explains the “I'll follow the minority” effect of self-expressive products. That is, findings revealed that low sales level will induce higher perceived self-image exclusivity, thereby weaken individuals' perceived face threat, and ultimately increase the intention to purchase the self-expressive products. Conversely, high (vs. low) sales levels will reduce perceived self-image exclusivity and hence consumers' perceived face threat, ultimately undermining the purchase intention (Study 2). More importantly, when discounting the belief that “high sales means commonplace and negative evaluations from others,” individuals' negative attitude toward the high sales level of self-expressive products will be eliminated. That is, when discounting the belief, there was no significant difference between consumers' purchase intentions toward self-expressive products with high and low sales level, and there was a significant difference between the high sales level group whose belief was discounted and the high sales level group whose belief was not discounted (Study 3).

From a theoretical perspective, the present study considered the sales level as a contextual cue that triggered the naive theory of exclusivity. The study extended the work of Steinhart et al.(Steinhart et al., 2014) that which one of naive theories a product induced depends on whether the product expresses users' self-image, focusing on the influence of self-expressive products' sales level on purchase intention. Different from the previous explanation from the perspective of individuals' uniqueness (Steinhart et al., 2014), this study explained the naive theory (Furnham, 1988; Deval et al., 2013) from the perspective of perceived face threat for the first time and provided evidence that the sales level information will affect perceived self-image exclusivity and then affect perceived face threat, which influences consumers' purchase intentions. In addition, a new perspective was proposed about online consumer behavior, namely, face threat, which has been investigated in social media (Oeldorf-Hirsch et al., 2017) but should also receive attention in online consumption research.

From a managerial perspective, the findings help marketers consider two trends. First, when promoting new self-expressive products on online retail websites, marketers should display self-expressive products with lower sales level, rather than best sellers, on the home page of recommendations; conversely, the functional products recommended there should have higher sales level. Second, considering a good way to change consumers' belief that “high sales means commonplace and negative evaluations from others” (e.g., by adding descriptions to increase the product exclusivity or decrease the possibility of face threat) will be an opportunity for the further promotion of products that already sell well.

Despite these advances, future research should also consider the impact of other factors on the conclusions of the present study. Such factors might include product price and brand reputation. For example, a consumer might buy an expensive self-expressive product with high sales level because that person might be proud to use an expensive product. Similarly, a famous brand will be significant factor that influences the effect of sales levels on the consumer's perception and purchase intention of self-expressive products, that is, when perceiving a self-expressive product with high sales level to be a famous brand, consumers might buy it because it is a brand with high prestige which will help them signal their identity. It may also be interesting to explore characteristics of individuals as a possible moderator. Specifically, individuals with stronger (vs. weaker) face consciousness will pay more attention to the sales level of self-expressive products, which will create an opportunity for online retail websites to make personalized recommendations for different individuals. Besides, all subjects in the present research are young consumers, whether the research conclusion is applicable to older consumer groups remains to be tested in the future.

## Author contributions

XW study design, data collection, data analysis, paper revising. TW study design, data collection, data analysis, paper writing, paper revising. JW study design, data analysis, paper revising.

### Conflict of interest statement

The authors declare that the research was conducted in the absence of any commercial or financial relationships that could be construed as a potential conflict of interest.
