# Supplementary material for: I'll Follow the Minority: The Effects of Sales Level on Purchase Intention of Self-expressive Products
Source: Front Psychol. 2018 Jul 10;9:1135. doi: 10.3389/fpsyg.2018.01135 (PMC6048468; doi:10.3389/fpsyg.2018.01135)
Supplement: Supplementary file 1 [file Table_1.docx]

**APPENDIX A: MEASURES USED IN STUDIES 1A, 1B, 2, AND 3**

| **Items** | **Notes** |
| --- | --- |
| **Status signalling capability** (1 = “*strongly disagree*” , 7 = “*strongly agree*”)  The product can convey one’s personality to the people around him/her.  The product reflect the owner's identity.  The product shape the owner's image in the eyes of others.  The product will tell others what kind of person the owner is. | Studies 1A, 1B and 2 |
| **Purchase intention** (1 = “*not at all*” , 7 = “*very much*”)  How likely would you be to buy the fashion coat (or heating blanket)? | Studies 2, 3 |
| **Perceived self-image exclusivity** (1 = “*strongly disagree*” , 7 = “*strongly agree*”)  You infer that others around you will not have the same image as you when using the fashion coat (or heating blanket). | Studies 2, 3 |
| **Perceived face threat** (1 = “*strongly disagree*” , 7 = “*strongly agree*”)  Using the fashion coat (or heating blanket) will make me feel awkward.  Using the fashion coat (or heating blanket) will make me feel embarrassed.  Using the fashion coat (or heating blanket) will make me feel uncomfortable.  Using the fashion coat (or heating blanket) will make me feel flustered.  Using the fashion coat (or heating blanket) will make me feel uneasy.  Using the fashion coat (or heating blanket) will make me feel exposed.  Using the fashion coat (or heating blanket) will make me look bad. | Studies 2, 3 |
| **Perceived uniqueness** (1=“*very low*”，7=“*very high*”)  The extent to which others think the product reflects its user's uniqueness. | Studies 1, 2 |
| **Sales level perception** (1 = “*strongly disagree*” , 7 = “*strongly agree*”)  Many others are likely to own this product.  Few others are likely to own this product. | Studies 1A, 1B, 2 and 3 |

**APPENDIX B: RESULTS OF STUDY 3**

|  | **Model 1** | | | | | | | | | | | | | | | | | | | |  |
| --- | --- | --- | --- | --- | --- | --- | --- | --- | --- | --- | --- | --- | --- | --- | --- | --- | --- | --- | --- | --- | --- |
|  | **M1 (perceive self-image exclusivity)** | | | | | | | **M2 (perceived face threat)** | | | | | | **Y (purchase intention)** | | | | | | |  |
|  | ***Coefficient*** | | | ***SE*** | | | | ***Coefficient*** | | ***SE*** | | | | ***Coefficient*** | | | | | | ***SE*** |  |
| X(high sales level with baseline belief =1; low sales level with baseline belief=0) | -2.44*** | | | 0.24 | | | | -0.05 | | 0.25 | | | | -0.04 | | | | | | 0.41 |  |
| M1 (perceive self-image exclusivity) | — | | | — | | | | -0.23* | | 0.10 | | | | 0.08 | | | | | | 0.12 |  |
| M2 (perceived face threat) | — | | | — | | | | — | | — | | | | -0.48*** | | | | | | 0.11 |  |
| Constant | 5.68** | | | 1.83 | | | | 2.47 | | 2.03 | | | | 2.40 | | | | | | 0.40 |  |
| Covariates | | | | | | | | | | | | | | | | | | | | |  |
| Perceived uniqueness | 0.28* | | | 0.09 | | | | -0.26* | | 0.10 | | | | 0.14 | | | | | | 0.12 |  |
| Age | -0.04 | | | 0.09 | | | | 0.19 | | 0.10 | | | | 0.18 | | | | | | 0.11 |  |
| Gender(male=1, female=2) | -0.61* | | | 0.23 | | | | -0.06 | | 0.25 | | | | -0.07 | | | | | | 0.29 |  |
|  | | | | | | | | | | ***Effect*** | | | | ***SE*** | | | ***LLCI*** | | | ***ULCI*** |  |
| Indirect effect of sales level on purchase intention through perceived self-image exclusivity | | | | | | | | | | -0.20 | | | | 0.30 | | | -0.8531 | | | 0.3130 |  |
| Indirect effect of sales level on purchase intention through perceived self-image exclusivity and perceived face threat in serial | | | | | | | | | | -0.27 | | | | 0.15 | | | -0.7130 | | | -0.0526 |  |
| Indirect effect of sales level on purchase intention through perceived face threat | | | | | | | | | | -0.02 | | | | 0.18 | | | -0.3688 | | | 0.3551 |  |
| Total effect of sales level on purchase intention | | | | | | | | | | -0.49 | | | | 0.35 | | | -1.2694 | | | 0.1190 |  |
|  | | | **Model 2** | | | | | | | | | | | | | | | | | |  |
|  | | | **M1 (perceive self-image exclusivity)** | | | | | | **M2 (perceived face threat)** | | | | | | **Y (purchase intention)** | | | | | |  |
|  | | | ***Coefficient*** | | | ***SE*** | | | ***Coefficient*** | | | ***SE*** | | | ***Coefficient*** | | | | | ***SE*** |  |
| X (high sales level with baseline belief =1; high sales level with discount belief=0) | | | -1.21*** | | | 0.28 | | | 0.46 | | | 0.25 | | | -0.66* | | | | | 0.30 |  |
| M1 (perceive self-image exclusivity) | | | — | | | — | | | -0.30*** | | | 0.08 | | | 0.26 | | | | | 0.10 |  |
| M2 (perceived face threat) | | | — | | | — | | | — | | | — | | | -0.33** | | | | | 0.11 |  |
| Constant | | | 4.67* | | | 2.16 | | | 2.69 | | | 1.80 | | | -0.70 | | | | | 2.2 |  |
| Covariates | | | | | | | | | | | | | | | | | | | | |  |
| Perceived uniqueness | | | -0.18 | | | 0.10 | | | -0.26* | | | 0.10 | | | 0.38** | | | | | 0.12 |  |
| Age | | | 0.12 | | | 0.10 | | | 0.19 | | | 0.10 | | | 0.32** | | | | | 0.17 |  |
| Gender(male=1, female=2) | | | 0.34 | | | 0.25 | | | -0.06 | | | 0.25 | | | -0.30 | | | | | 0.30 |  |
|  | | | | | | | | | | | | ***Effect*** | | | ***SE*** | | ***LLCI*** | | | ***ULCI*** |  |
| Indirect effect of high sales level with different belief on purchase intention through perceived self-image exclusivity | | | | | | | | | | | | -0.03 | | | 0.10 | | -0.2455 | | | 0.1597 |  |
| Indirect effect of high sales level with different belief on purchase intention through perceived self-image exclusivity and perceived face threat in serial | | | | | | | | | | | | -0.12 | | | 0.07 | | -0.3292 | | | -0.0304 |  |
| Indirect effect of high sales level with different belief on purchase intention through perceived face threat | | | | | | | | | | | | -0.15 | | | 0.10 | | -0.3988 | | | -0.0169 |  |
| Total effect of high sales level with different belief on purchase intention | | | | | | | | | | | | -0.31 | | | 0.15 | | -0.6568 | | | -0.0692 |  |
|  | | **Model 3** | | | | | | | | | | | | | | | | | | | |
|  | | **M1 (perceive self-image exclusivity)** | | | | | **M2 (perceived face threat)** | | | | | | **Y (purchase intention)** | | | | | | | | |
|  | | ***Coefficient*** | | | ***SE*** | | ***Coefficient*** | | | | ***SE*** | | ***Coefficient*** | | | | | ***SE*** | | | |
| X (high sales level with discount belief =1; low sales level with baseline belief=0) | | -1.34*** | | | 0.26 | | -1.41*** | | | | 0.22 | | 0.25 | | | | | 0.27 | | | |
| M1 (perceive self-image exclusivity) | | — | | | — | | -0.143* | | | | 0.22 | | -0.03 | | | | | 0.07 | | | |
| M2 (perceived face threat) | | — | | | — | | — | | | | — | | -0.16 | | | | | 0.10 | | | |
| Constant | | 5.31* | | | 2.21 | | 1.79 | | | | 1.71 | | -1.41 | | | | | 1.81 | | | |
| Covariates | | | | | | | | | | | | | | | | | | | | | |
| Perceived uniqueness | | 0.22 | | | 0.13 | | -0.23* | | | | 0.10 | | 0.35** | | | | | 0.10 | | | |
| Age | | -0.04 | | | 0.12 | | 0.19 | | | | 0.90 | | 0.30 | | | | | 0.10 | | | |
| Gender(male=1, female=2) | | -0.04 | | | 0.12 | | 0.06 | | | | 0.22 | | 0.01** | | | | | 0.23 | | | |
|  | | | | | | | | | | | ***Effect*** | | ***SE*** | | | ***LLCI*** | | | ***ULCI*** | | |
| Indirect effect of sales level on purchase intention through perceived self-image exclusivity | | | | | | | | | | | 0.04 | | 0.10 | | | -0.1525 | | | 0.2504 | | |
| Indirect effect of sales level on purchase intention through perceived self-image exclusivity and perceived face threat in serial | | | | | | | | | | | -0.31 | | 0.03 | | | -0.1276 | | | 0.0013 | | |
| Indirect effect of sales level on purchase intention through perceived face threat | | | | | | | | | | | 0.23 | | 0.15 | | | -0.0577 | | | 0.5552 | | |
| Total effect of sales level on purchase intention | | | | | | | | | | | 0.24 | | 0.19 | | | -0.1177 | | | 0.6083 | | |

*Note: number of bootstrap resamples = 5000;* *∗p < 0.05; ∗∗p < 0.01; ∗∗∗p < 0.001.*
